# Focusing Points on FSCJ’s Guideline Recently Established: Risk Assessment of Food Contact Materials

**DOI:** 10.14252/foodsafetyfscj.D-21-00029

**Published:** 2022-06-24

**Authors:** Masahiro Nakamoto

**Affiliations:** Food Safety Commission Secretariat, Cabinet Office, Government of Japan, 5-2-20 Akasaka, Minato-ku, Tokyo 107-6122, Japan

**Keywords:** Key words food apparatus, containers, and packaging, FCMs, risk assessment, guidelines, tiered approach, TTC

## Abstract

In Japan, the Positive List (PL) system was introduced (Enforcement: June 1, 2020) in the regulative field of Food Apparatus, Containers, and Packaging (ACP) by the recent amendment of the Food Sanitation Act. Under this situation, continuous requests for the risk assessments from the Ministry of Health, Labour and Welfare (MHLW) to the Food Safety Commission of Japan (FSCJ) will be expected. To enhance fairness and transparency and to clarify the data required for the risk assessment, the FSCJ established its “Guidelines for the Risk Assessment of Food Apparatus, Containers, and Packaging” on May 28, 2019. The Guidelines apply to new Food Contact Materials or Substances (FCMs) after enforcement of the PL system (June 1, 2020). The subject material is synthetic resins, because the PL system was first introduced to them in Japan. In general, the substances that are migrated from ACP are not intended to migrate into foods, and their technological effects on foods are not expected. It can be supposed that the migration of these substances is generally very limited. Therefore, as adopted in the USA and the EU, the Guidelines also adopt the tiered approach for the toxicological data requirement that depend on the estimated migration levels (Tier of Dietary Concentration (Tier of DC)) on the subject substance. The greater the exposure to the substance through migration, the more toxicity test results will be needed. The risk assessment steps by the tiered approach in the Guidelines are (1) migration assessment, (2) toxicity assessment, (3) exposure assessment, and (4) risk characterization. These steps are aimed to harmonize with the general 4 steps of risk assessments: hazard identification, hazard characterization, exposure assessment, and risk characterization. In this review, we will introduce the overview of the Guidelines and details of the above 4 steps.

## 1. Introduction

In Japan, the Ministry of Health, Labour and Welfare (MHLW), one of the risk management agencies, is implementing risk management on Food Apparatus, Containers, and Packaging (ACP) under the Food Sanitation Act (act No. 233, 1947). Conventionally, to ensure the safety of ACP, risk management was implemented only by the Negative List system where the use of Food Contact Materials and Substances (FCMs) for ACP was restricted only when some standards and criteria under the Food Sanitation Act were established on those FCMs. Recently, however, the Positive List (PL) system was introduced (Promulgation: June 16, 2018. Enforcement: June 1, 2020) in the regulative field of ACP by the amendment of the Food Sanitation Act, which attempts to respond to changes in food environment and globalization. In the PL system, the use of FCMs is principally restricted unless FCMs are ensured to be safe and listed in the PL of Standards and Criteria for Food and Food Additives, etc. (Public Notice of Ministry of Health and Welfare No. 370, 1959).

In Japan, under the Food Safety Basic Act (Act No. 48, 2003), requests for the risk assessment to Food Safety Commission of Japan (FSCJ) are mandatory when the standards and criteria of the ACP will be amended. Continuous requests for the risk assessments from the MHLW to the FSCJ will be, therefore, expected by the introduction of the PL system. This will highlight the increased importance of enhancing fairness and transparency and should clarify the data required for the risk assessment. The FSCJ considered the approach for the risk assessment of ACP comparing those of internal/external of Japan and established “Guidelines for the Risk Assessment of Food Apparatus, Containers, and Packaging (hereinafter refer to as the Guidelines)” on May 28, 2019. Based on the Guidelines, the FSCJ will conduct the risk assessment of the new FCMs that will be in use after the enforcement of the PL system. In this review, we introduce the summary of the Guidelines.

## 2. Overview of the Guidelines

### 2.1. Purpose

The purpose of the Guidelines is to enhance the fairness and transparency of risk assessments of FCMs for ACP and to facilitate the deliberations on them in the FSCJ. To achieve this, the Guidelines clarify the policy, method, and data requirements for the risk assessments.

### 2.2. Scope

The Guidelines apply to the risk assessments of new FCMs. The subject materials of the Guidelines are synthetic resins, because the PL system was introduced to them in Japan. The synthetic resins for ACP will contain many kinds of substances such as raw materials, impurities derived from them, unintentionally contained substances through manufacturing processes, and these substances may migrate into the foods with which ACP comes in contact.

The subject substances of the Guidelines are, therefore, those that migrate from ACP into the foods. These are supposed to be the intentionally used substances (raw materials), or the unintentionally contained substances (impurities, byproducts, decomposition products).

### 2.3. Features

In general, the substances that are migrated from ACP are not intended to migrate into foods, and their technological effects on foods are not expected. It can be supposed that the migration of these substances is generally very limited and the consumption of these by humans is also. Therefore, to uniformly require all kinds of toxicological data such as the results of genotoxicity tests, repeated dose toxicity tests, reproductive and developmental toxicity tests, carcinogenicity tests, ADME (Absorption, Distribution, Metabolism, Excretion) studies for all substances will not relevant for the risk assessment of FCMs for ACP. As for the United States of America (USA) and the European Union (EU) where the PL system on FCMs was already introduced and risk assessment of these have been conducted, the toxicological data requirements depend on the levels of migration into foods that are estimated by the migration testing^1^^,^^2^^)^.

Considering the above, the Guidelines adopt the tiered approach for the toxicological data requirement that depend on the estimated migration levels (Tier of Dietary Concentration (Tier of DC)) on the subject substance. The greater the exposure to the substance through migration, the more toxicity test results will be needed. By comparison to the tiered approach in the USA and the EU, the boundary values of each Tier of DC are the same with USA’s (Table 1). The risk assessment steps by the tiered approach in the Guidelines are (1) migration assessment; (2) toxicity assessment; (3) exposure assessment; and (4) risk characterization. These steps are aimed to harmonize with the general four steps of risk assessments: hazard identification; hazard characterization; exposure assessment; and risk characterization.

**Table 1.**
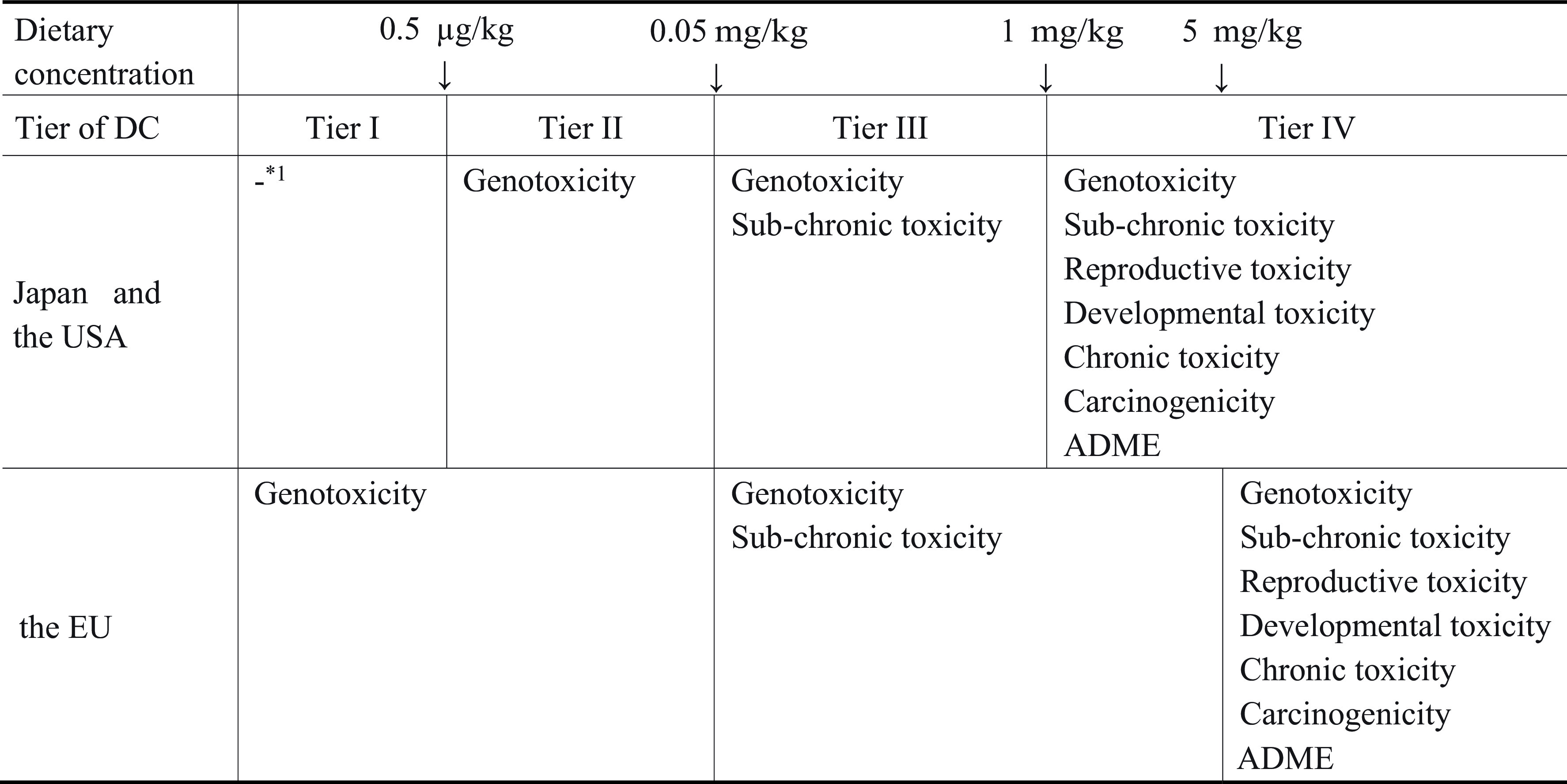
Tiered approach (test data requirement in each Tier of DC) among Japan, the USA, and the EU

*^1^ Available information on genotoxicity

## 3. Migration Assessment

### 3.1. Approaches in General

#### 3.1.1. Outline

For migration assessment, Tier of DC is decided by dietary concentration (DC) of the subject substance. DC is defined as the concentration of the subject substance in a daily unit of meal. DC is converted from the quantity of migration with some conversion factors that reflect usage and prevalence situations of manufacturing materials for ACP in Japan. The calculation formula adopted in the Guidelines is compatible to the situations in Japan and different from that of the USA and the EU, because the calculation formula will be depend on the usage and prevalence situations of ACP, and the risk management system in each country or region.

The quantity of migration is calculated by the data obtained from the migration testing. In migration testing, food simulants (solvents or substances that simulate the physical and chemical properties of specific food categories) are used because of the practical reason and the feasibility of analysis. As for the practical reason, there is a need to appropriately encompass and summarize the diversity of usage conditions of ACP (e.g. types of food, and temperature/time conditions in food contacting). On the feasibility of analysis, it is quite difficult in general to analyze and quantify the very low concentration of the subject substance in food, because of the complexity of components in the food matrix. For the above two reasons, food simulants other than foods themselves are appropriate, and the migration testing conditions are set also under the consideration for the calculation formula for DC.

#### 3.1.2. Features of the Migration Assessment

The migration testing conditions and the calculation formula for DC in the Guidelines were proposed based on the study supported by a research grant program of the FSCJ^3^^)^.

The testing conditions and the calculation formula are different from those of the USA and the EU, which reflects the differences of the PL system among Japan, the USA, and the EU (Table 2). For example, the regulations for additives are different. In the USA and Japan, the use of additives is regulated mainly by the use level. In the EU, that is regulated mainly by the migration level. The applicable scope of polymers is also different. In the USA, that is limited in the scope of the notification by the applicant. In Japan, that is limited in the scope of the synthetic resin groups (Table 3) relevant for the risk assessment. In the EU, there is basically no limitation if there is no specific consideration.

**Table 2.** The PL system for synthetic resin and calculation formula for dietary concentration among Japan, the USA, and the EU

		the USA (FCN^*^^1^)	Japan	the EU
The PL system				
Scope of substances		Substances or products that are notified by the applicant.	Base polymer^*2^ Additive	Monomer Additive
Approach for regulation (addition/elusion amount)		Depend on the notification by the applicant Additive Use level	Base polymer N.A Additive Use level	Monomer Migration level Additive Migration level
Applicable scope of polymer for additive		Depend on the notification by the applicant	Subject synthetic resin group	Basically no limitation if there is no specific consideration
		Narrow	→	Wide
Calculation formula for dietary concentration (DC)				
Scope of calculation for DC		Depend on submission	Synthetic resin group (In applying to multiple synthetic resin groups, total DC is used)	All types of synthetic resin
Conversion factors for DC	Consumption factor (CF)^*3^	Apply	Apply	Not apply
	Distribution factor (DF)^*4^	Apply	Apply	Not apply
	Reduction factor (RF)^*5^	Applicable depending on submission	Applicable	Applicable for oils, fats, and fatty foods
Calculation formula (Q: Migration amount in migration testing)		DC =∑(Q × DF) × CF× RF Q: 4 types depending on food category ∑: Total of the result of each food category	DC =∑(Q × DF) × CF× RF Q: 5 types depending on food category ∑: Accumulation of each food category DC_total_=∑ DC ∑: Accumulation of each synthetic resin group	DC = Q (× RF)
		Realistic	→	Conservative

*^1^ Food Contact Substance Notification Program

*^2^ Main structural component of the synthetic resin

*^3^ Factor that indicates the ratio of amount of meal contacted with a specific type of synthetic resin

*^4^ Factor that indicates the ratio of ACP used for a specific food category in a specific type of synthetic resin

*^5^ Factor used for lowering a value of predefined consumption factor or distribution factor

**Table 3.** Synthetic resin groups

Group	Description
Group 1	Group of polymers (exclude those falling under synthetic resin groups 4 to 7) that have either the Glass transition temperature or the Ball pressure temperature of 150°C and above, or crosslinking structure and melting point of 150°C and above
Group 2	Group of polymers (exclude those falling under synthetic resin group 1 and groups 4 to 7) with water absorption 0.1% or less
Group 3	Group of polymers (exclude those falling under synthetic resin group 1 and Groups 4 to 7) with water absorption more than 0.1%
Group 4	Group of polymers in which 50% or more are made from vinyl chloride or vinylidene chloride
Group 5	Group of polymers in which 50% or more are made from ethylene
Group 6	Group of polymers in which 50% or more are made from propylene
Group 7	Group of polymers in which 50% (mol %) or more are made from terephthalic acid and ethylene glycol

The calculation formula for DC in the Guidelines is similar to that of the USA. However, to accumulate DC among synthetic resin groups is one of the representative feature of the Guidelines because of the unique management systems in Japan where polymers are categorized into relevant synthetic resin groups.

### 3.2. Migration Testing

In principle, the immersion method is adopted as the migration testing method. In the immersion method, the test specimen, which includes the subject substances, is immersed to the food simulants. The test volume of food simulants is 1.5 – 2.0 mL per 1 cm^2^ of test specimen. The food simulants are corresponding to the food categories (Table 4). The relevant food simulants are selected according to the food categories that cover the foods of contacting ACP in the proposed usage conditions. If foods in subject are fit in the definitions of multiple food categories, the relevant food simulants are applied.

**Table 4.** Food categories and food simulants

	Food category		Food simulant
D_1_	Normal foods	Foods not falling under D_2_, D_3_, D_4_ and D_5_	Distilled water
D_1sub_	Dried foods	Foods of D_1_ with water content of 20% or less (weight %) within it or on the surface of it	PPO^*1^
D_2_	Acidic foods	Foods having a pH 4.6 or less within it or on the surface of it	4% acetic acid (volume %)
D_3_	Alcohols	Beverages with an alcohol content of 1% or more (volume %) within it or on the surface of it	20% ethanol (volume %)
D_4_	Milk and dairy products	Foods with fat content of less than 20% (weight %) within it or on the surface of it, among foods subject to Article 2 of Ministerial Ordinance on Milk and Milk products Concerning Compositional Standards, etc. (Ordinance of the Ministry of Welfare, No.52, 1951; hereinafter refer to as Ministerial Ordinance on Milks)	50% ethanol (volume %)
D_5_	Oils, fats, and fatty foods	Foods with fat content of 20% or more (weight %) within it or on the surface of it (including those are subject to Article 2 of Ministerial Ordinance on Milks and not falling under D_4_)	Vegetable oil^*2^

*^1^ Applicable when ACP are used only for dried foods (D_1sub_) among normal foods (D_1_)

*^2^ 95% ethanol (volume %), isooctane or heptane may be used in place of vegetable oil.

There are two temperature/time conditions for the migration testing; High Temperature/Short period of Time (HTST) and Low Temperature/Long period of Time (LTLT) (Table 5). For HTST, the relevant one of three temperature/time conditions is selected based on the information of food contacting temperature of ACP as the proposed usage conditions. The food contacting temperature is divided into three ranges (70°C or less, more than 70°C and up to 100°C, and more than 100°C) and the relevant one that covers the proposed usage condition is selected. If the thermal register temperature of the synthetic resin is below the default temperature conditions, the thermal register temperature can be selected as the temperature condition instead of the default one. For LTLT, the temperature/time condition is 40°C /10 days, regardless of the food contacting temperature. This condition will be expected to cover the long time contacting condition of ACP. Therefore, if the contacting time is limited to 30 minutes, the migration testing on LTLT can be omitted.

**Table 5a.** Temperature/time conditions for migration testing

Food category	Food simulant	Types of synthetic resin^*2^	Temperature/time conditions^*1^			
			High temperature/Short period of time			Low temperature/ Long period of time
			Food contacting temperature			
			More than 100°C	More than 70°C and up to 100°C	70°C or less	
D_1_	Distilled water	All	120°C / 30 min	90°C / 30 min	60°C / 30 min	40°C / 10 d
D_1sub_	PPO	All	120°C / 30 min	90°C / 30 min	60°C / 30 min	40°C / 10 d
D_2_	4% acetic acid	All	90°C / 4 h	90°C / 30 min	60°C / 30 min	40°C / 10 d
D_3_	20% ethanol	All	60°C / 2 d	60°C / 6 h	60°C / 30 min	40°C / 10 d
D_4_	50% ethanol	G1G2 (Exclude PS)G3 (Exclude PA)	60°C / 2 d	60°C / 6 h	60°C / 30 min	40°C / 10 d
		PSPAPET	60°C / 2 d	60°C / 60 min	40°C / 30 min	30°C / 10 d
		Other than those above	60°C / 12 h	60°C / 60 min	40°C / 30 min	30°C / 10 d
D_5_	Vegetable oil	All	120°C / 30 min	90°C / 30 min	60°C / 30 min	40°C / 10 d

**Table 5b.** Temperature/time conditions for migration testing (continued)

Food category	Food simulant	Types of synthetic resin^*2^	Temperature/time conditions^*1^			
			High temperature/Short period of time			Low temperature/ Long period of time
			Food contacting temperature			
			More than 100°C	More than 70°C and up to 100°C	70°C or less	
Alternative solution (95% ethanol, isooctane or heptane) to a vegetable oil						
	95% ethanol	PE	60°C / 2 d	60°C / 4 h	40°C / 30 min	40°C / 10 d
		PP	60°C / 2 d	60°C / 4 h	60°C / 30 min	40°C / 5 d
		PET	60°C / 4 h	-	-	-
		PVC	60°C / 90 min	-	-	-
		PVDC	60°C / 4 h	60°C / 30 min	40°C / 30 min	30°C / 5 d
		PS	60°C / 1 d	60°C / 90 min	40°C / 30 min	20°C / 2 d
		PA	-	-	-	-
		Others	60°C / 2 d	60°C / 4 h	60°C / 30 min	40°C / 10 d
	Isooctane	PE	60°C / 90 min	60°C / 30 min	40°C / 30 min	20°C / 2 d
		PP	60°C / 90 min	60°C / 30 min	40°C / 30 min	20°C / 2 d
		PET	-	60°C / 12 h	40°C / 30 min	40°C / 5 d
		PVC	60°C / 1 d	60°C / 90 min	40°C / 30 min	30°C / 10 d
		PVDC	60°C / 1 d	60°C / 90 min	40°C / 30 min	40°C / 5 d
		PS	60°C / 90 min	-	-	20°C / 2 d
		PA	60°C / 2 d	60°C / 90 min	40°C / 30 min	30°C / 5 d
		Others	60°C / 2 d	60°C / 12 h	40°C / 30 min	40°C / 5 d
	Heptane	PE	60°C / 90 min	-	-	-
		PP	60°C / 90 min	-	-	-
		PET	60°C / 1 d	60°C / 30 min	40°C / 30 min	20°C / 5 d
		PVC	60°C / 4 h	60°C / 30 min	40°C / 30 min	20°C / 2 d
		PVDC	60°C / 4 h	60°C / 30 min	40°C / 30 min	20°C / 10 d
		PS	-	-	-	-
		PA	60°C / 2 d	60°C / 90 min	40°C / 30 min	30°C / 5 d
		Others	60°C / 2 d	60°C / 90 min	40°C / 30 min	30°C / 5 d

*^1^ “-” indicates that no applicable temperature/time conditions are provided.

*^2^  PE (polyethylene), PP (polypropylene), PET (polyethylene terephthalate), PVC (polyvinyl chloride), PVDC (polyvinylidene chloride), PS (polystyrene), PA (polyamide). G1, G2, G3 indicate synthetic resins correspond to those in the synthetic resin Group 1, Group 2 and Group 3, respectively.

### 3.3. Analysis on Chemical Substances in Food Simulants

The analytical equipment is required to have appropriate detection principle that covers the physical and chemical properties of the substances that are assumed to migrate into food simulants (hereinafter referred to as “the target substances”). The method of analysis is required to have appropriate sensitivity, and to be validated or verified.

For the assessment on additives, the target substances will be the additives themselves. For the assessment on a base polymer (main structural component of the synthetic resin), the target substances will be constituent monomers of the base polymer. Other than theses, impurities, byproducts, decomposition products can be also included in the target substances if the structure of these substances was already identified and the possibility of migration into food simulants is supposed to be clearly high.

When analyzing food simulants, the detection of the target substances is examined first. In this step, non-target substances may also be detected. The estimation or the identification of non-target substances might be, therefore, also necessary if these substances are supposed to be derived from the additive or the base polymer in subject, and the estimation or identification is reasonably feasible under the general levels of analytical technology. In the Guidelines, the general scope of the subject substances is defined as the substances that migrate from ACP into the foods. In more detail, the target substances and non-target substances that are detected and reasonably estimated or identified are regarded as the subject substances in the actual risk assessment. For these subject substances, the concentration in food simulants (µg/mL) is quantified.

### 3.4. Quantity of Migration

Quantity of migration (“q (mg/kg)”) is calculated by formula 1 using the some parameters.

q=(C×V×600)÷1000 (Formula 1)

“C (µg/mL)” is the concentration in food simulants quantified the above migration testing. “V (mL/cm^2^)” is the volume of food simulants per unit of contacting surface of specimen. “600 (cm^2^/kg)” is the factor for converting from contacting surface to contacting food weight. This is based on the hypothesis that the 1 kg of foods are contained in a cubic container with each side of 10 cm (Inner surface area is 600 cm^2^). “1000” is the factor for converting from µg to mg. After the above calculation, quantity of migration between HTST and LTLT in each food category are compared and the bigger value one is adopted as the maximum quantity of migration (“Q (mg/kg)”) in each food category.

### 3.5. Dietary Concentration and Its Tier Levels

Dietary concentration (“DC (mg/kg diet)”) is calculated using the maximum quantity of migration (“Q (mg/kg)”) and other conversion factors; consumption factor (“CF”), distribution factor (“DF”), and reduction factor (“RF”).

CF is set in each synthetic resin group, and DF is set in each food category (Table 6). These conversion factors reflect usage and prevalence of manufacturing materials for ACP in Japan. For example, CF of synthetic resin group 5 (polyethylene) is 0.25. This means that the occupation rate of polyethylene among the essential ACP materials (such as synthetic resin (including its type), paper, metals) in the marketplace is 25%. DF of the food category D_5_ (oils and fats, and fatty foods) for the synthetic resin group 5 (polyethylene) is 0.05. This means that the occupation rate of oils and fats, and fatty foods among all food categories that are contact with polyethylene in market place is 5%. RF can be set from 0.2 to 0.8 and multiplied by CF and/or DF as necessary, when the usage and prevalence of manufacturing materials are expected to be limited.

**Table 6.** Consumption factor (CF) and distribution factor (DF)

Synthetic resin groups (Types of synthetic resin^*^^1^)	CF	DF					
		Normal foods		Acidic foods	Alcohols	Milks^*2^	Oils^*3^
			Dried foods				
		D_1_	D_1sub_	D_2_	D_3_	D_4_	D_5_
Group 1	0.05	DF for the food category with the largest maximum quantity of migration (Q) is 0.96, and DF for other food categories are 0.01.					
Group 2 (PS and other resins falling under Group 2)	0.07	0.38	0.02	0.27	0.01	0.11	0.23
		The case FCMs are not used for manufacturing PS DF for the food category with the largest maximum quantity of migration (Q) is 0.96, and DF for other food categories are 0.01.					
Group 3 (PA and other resins falling under Group 3)	0.05	0.92	0.01	0.01	0.01	0.01	0.05
		The case FCMs are not used for manufacturing PA DF for the food category with the largest maximum quantity of migration (Q) is 0.96, and DF for other food categories are 0.01.					
Group 4 (PVC, PVDC)	0.05	0.93	0.01	0.01	0.01	0.01	0.04
Group 5 (PE)	0.25	0.88	0.03	0.04	0.01	0.02	0.05
Group 6 (PP)	0.16	0.80	0.05	0.05	0.01	0.02	0.12
Group 7 (PET)	0.22	0.86	0.01	0.09	0.01	0.01	0.03

*^1^ PS (polystyrene), PA (polyamide), PVC (polyvinyl chloride), PVDC (polyvinylidene chloride), PE (polyethylene), PP (polypropylene), PET (polyethylene terephthalate).

*^2^ Milk and dairy products (exclude those falling under oils, fats and fatty foods)

*^3^ Oils, fats and fatty foods

In the Guidelines, “DC (mg/kg diet)” is calculated by formula 2, here food category is D_i_ (i = 1, 2, 3, 4, 5) and maximum quantity of migration and DF of each food category are expressed as “Q_i_ (mg/kg)” and “DF_i_” respectively.

$$\begin{array}{c} \text{DC}=\left(\overset{5}{\underset{\text{i}=1}{\sum }}\left({\text{Q}}_{\text{i}}\times {\text{DF}}_{\text{i}}\right)\right)\times \text{CF}\\ {\text{* As necessary, RF can be multiplied to CF and/or DF}}_{\text{i}}\text{.} \end{array}$$ (Formula 2)

In formula 2, “DC (mg/kg diet)” is calculated for only the single synthetic resin group. The total DC (“DC_toral_ (mg/kg diet)”) among all synthetic resin groups is calculated by formula 3, here synthetic resin groups number is j (j =1, 2, 3, 4, 5, 6, 7) and DC of each synthetic resin group is expressed as “DC_j _(mg/kg diet)”.

$${\text{DC}}_{\text{total}}=\overset{7}{\underset{\text{j}=1}{\sum }}{\text{DC}}_{\text{j}}$$ (Formula 3)

DC calculated by formula 3 is compared with the range of DC designated for each Tier of DC (Table 7), and Tier of DC on the subject substance is finally decided.

**Table 7.** Tier of DC and range of dietary concentration

Range of dietary concentration				Tier of DC
		0.5 µg/kg	at or less	Tier I
0.5 µg/kg	above	0.05 mg/kg	at or less	Tier II
0.05 mg/kg	above	1 mg/kg	at or less	Tier III
1 mg/kg	above			Tier IV

## 4. Toxicity Assessment

### 4.1. Approaches in General

The toxicity of the subject substances is examined based on the results of toxicity tests that are designated to each Tier of DC and other relevant available information. If necessary, the Health Based Guidance Value (HBGV) is set.

There are 4 levels of Tier of DC, from Tier I where no toxicity test is required, to Tier IV, where full sets of toxicity tests including carcinogenic, reproductive and developmental toxicity tests are required (Table 1). The boundary levels between these Tiers were set under the consideration for the concept of Toxicological Threshold of Concern (TTC). The concept of TTC is based on the idea that, for the small amount of substances like in foods, there can be a general threshold level of exposure where the possibility of human health concern is considered to be very low. TTC have been examined and set for each type of chemicals that are categorized by the suggested levels of toxicity assumed by features of the chemical structures^4^^)^. First, we introduce the boundary level between Tier II and III, because one of the representative TTC that were proposed by Munro et al. (1996)^5^^)^ is applied.

### 4.2. Boundary Level Between Tiers II and III

#### 4.2.1. Setting Basis

The boundary level between Tiers II and III (0.05 mg/kg diet) is based on the TTC proposed by Munro et al. (1996) for chemicals that are classified into class III of Cramer structural classification. Munro et al. (1996) reported the analytical results of non-carcinogenic effects for each class of Cramer structural classification chemicals by analyzing the dataset on 613 substances such as industrial chemicals, medicines, and food ingredients. Cramer structural classification were proposed by Cramer et al. (1978)^6^^)^. This classification method is based on the potential tendency for metabolism which is assumed by features of the chemical structure. Chemicals are classified into class I, class II, or class III, and their suggested toxicity is assumed to be greater in this order (Table 8). Class III is, therefore, suggested to have greatest toxicity among these classes. Munro et al. (1996) analyzed 448 chemicals that were classified into class III. The 5 percentile value of distribution curve on No-Observed-Effect Level (NOEL) was obtained by analyzing the results of typical toxicity tests (sub-chronic, chronic, reproductive and developmental toxicity tests). After the uncertainty factor 100 was applied to this 5 percentile NOEL values, the TTC was set as 0.09 mg/person/day.

**Table 8.** Cramer structural classification

Cramer Class	Description
Class I	Substances with simple chemical structures and for which efficient modes of metabolism exist, suggesting a low order of oral toxicity.
Class II	Substances which possess structures that are less innocuous than class I substances, but do not contain structural features suggestive of toxicity like those substances in class III.
Class III	Substances with chemical structures that permit no strong initial presumption of safety or may even suggest significant toxicity or have reactive functional groups.

Although the other articles^7^^,^^8^^,^^9^^,^^10^^,^^11^^,^^12^^)^ including the article whose study subject is substances that are used for synthetic resins for ACP reported different TTC values on class III chemicals, the TTC proposed by Munro et al. (1996) is generally more conservative. The FSCJ, therefore, converted the TTC (0.09 mg/person/day) proposed by Munro et al. (1996) into DC (0.05 mg/kg diet) with the assumption of 2 kg diet/person/day, and adopted this value as the boundary level of Tiers II and III.

#### 4.2.2. Interpretation

The TTC value proposed by Munro et al. (1996) is assumed to be threshold for non-carcinogenic toxicity, and thus if DC of the subject substances below or at this boundary value (0.05 mg/kg diet) and the substance is not genotoxic, the concern for its non-carcinogenic toxicity and non-genotoxic carcinogenicity would be low. Accordingly, when DC is below or at this boundary value (Tier I or II), toxicity assessment could be focused on genotoxicity. On the other hand, when DC exceeds this boundary value (Tier III or IV), toxicities other than genotoxicity should also be assessed.

### 4.3. Boundary Level Between Tiers I and II

#### 4.3.1. Setting Basis

The boundary level between Tiers I and II (0.5 µg/kg diet) is based on the Virtually Safe Dose (VSD), which is extrapolated from TD_50_ (the dose of 50% incidence of cancer) obtained from data on the cancer incidence by carcinogens. According to the U.S. Food and Drug Administration (USFDA), when DC of food contact substances that will or might transfer to foods is below the level of Threshold of Regulation (TOR), the substances can be applied to TOR and excluded from the regulation on additives. The level of TOR is 0.5 µg/kg diet^13^^)^, and the boundary level between Tiers I and II is consistent to this value. In setting the level of TOR, Munro (1990)^14^^)^ was referred. Munro (1990) analyzed the distribution of VSD (the dose of 10^-6^ or 10^-5^ risk of cancer) using the data on liner extrapolations of TD_50_ on dose-response curves of 343 carcinogens. Although Munro (1990) concluded that 1 µg/kg diet may be relevant for the levels of TOR, the USFDA adopted 0.5 µg/kg diet to ensure the protection of public health^15^^)^.

#### 4.3.2. Interpretation

As mentioned above, toxicity assessment should be focused on genotoxicity, when Tier of DC is Tier I or II. If DC is below or at the boundary level between Tiers I and II (0.5 µg/kg diet), the lifetime cancer risk will be below or at 10^-6^ when there is no concern for genotoxicity, even if the subject substance is a carcinogen. Accordingly, for Tier I, the available information (e.g. structure-reactivity relationships) for the genotoxicity of the subject substance is required for the toxicity assessment, and the genotoxicity test results are not always necessary. For Tier II, the genotoxicity test results are required for the toxicity assessment.

### 4.4. Boundary Level Between Tiers III and IV

#### 4.4.1. Setting Basis

The boundary level between Tiers III and IV (1 mg/kg diet) is based on some scientific evidence for chronic, reproductive and developmental toxicities. In setting this level, the tiered approach of FCM risk assessment in the USA and the EU^1^^,^^2^^)^ was also referred. In the USA and the EU, full sets of toxicity tests are required, when DC (in the EU, migration concentration for food) is above 1 mg/kg diet and 5 mg/kg food, respectively (Table 1).

Barlow (1994)^16^^)^ reported that by referring to the scientific evidence for chronic, reproductive and developmental toxicities, the effects of these toxicities would be unlikely to occur, when the exposure level is below or at 0.1 mg/kg bw/day. The author also reviewed that intakes from food would not excess 0.1 mg/kg bw/day, if the migration range is 0.05 – 5 mg/kg food. Van Ravenzwaay et al. (2017)^17^^)^ reported that the TTC values that are obtained from the analysis of dataset of developmental toxicity tests (mainly OECD TG414) for industrial chemicals are 100 µg/kg bw/day and 95 µg/kg bw/day in rats and rabbits, respectively. The FSCJ converted these TTC to DC and obtained approximately 3 mg/kg diet with the assumption that the body weight is 60 kg and dietary consumption of foods is 2 kg for human adults. Frawley (1967)^18^^)^ reported that by analyzing the 2 year chronic toxicity tests for 220 chemicals (e.g. food additives, industrial chemicals, chemicals for food packaging, pesticides, heavy metals), only 5 substances (all of them are pesticides) indicate some effects when the exposure level is below or at 1 mg/kg diet.

Considering the above evidence and information conservatively, the FSCJ adopted 1 mg/kg diet for boundary level between Tiers III and IV.

#### 4.4.2. Interpretation

For Tiers III and IV, it is not relevant that the concern for non-carcinogenicity and non-genotoxic carcinogenicity would be generally low, and more attention should be paid for Tier IV in terms of exposure levels. Accordingly, for Tier IV, full sets of toxicity test results are required for the toxicity assessment. For Tier III, the toxicity test results of screening levels (genotoxicity and sub-chronic toxicity test results) are required for the toxicity assessment.

### 4.5. Toxicological Effects and Substances that Require Special Consideration

As explained in 4.2., the TTC value proposed by Munro et al. (1996) is adopted as the boundary level between Tiers II and III. This TTC was set from the 5 percentile value of distribution curve on NOEL by analyzing the subject dataset. Deriving from this, there are the following two limitations for the application of this TTC:

(1) First limitation is the type of toxicological effects. This TTC is not applicable to the toxicological effects that might occur at the exposure level below this 5 percentile value (e.g. neurotoxicity, toxicity derived from bioaccumulation).

(2) Second limitation is the type of substance. This TTC is not applicable to substances that were not included in the subject dataset (e.g. metals, inorganic compounds).

Accordingly, additional requirements were set about toxicological effects and substances to which the TTC is not applicable (Table 9), considering relevant evidence and information on TTC approach^4^^,^^12^^,^^19^^,^^20^^,^^21^^)^ and assessment guidance in the USA and the EU^1^^,^^2^^)^.

**Table 9.** Toxicological effects and substances that are required special consideration

Toxicological effects that are required special consideration	
Neurotoxicity, Immunotoxicity Toxicity derived from endocrine activity	• Regardless of Tier of DC, relevant test results might be required if these toxicological effects are suspected by available information.
Toxicity derived from bioaccumulation	
Tier I or II	• Relevant test results might be required for the following substances. - Substances that are considered to be highly bioaccumulative (e.g. polyhalogenateddibenzo-p-dioxins, polyhalogenated dibenzofuran, polyhalogenated biphenyls) - Substances that are judged through the consideration on log P_ow_ (Octanol/water partition coefficient) and other special concerns about bioaccumulation (e.g. chemical structure)
Tier III	Log P_ow_< 3 • Relevant test results other than sub-chronic toxicity test result might be required when there are special concerns about bioaccumulation (e.g. chemical structure). Log P_ow_≥ 3 • Relevant test results other than sub-chronic toxicity test result would be required.
Tier IV	• ADME study results are used for consideration on bioaccumulation.
Substances that are required special consideration	
Metals, Inorganic compounds, Proteins	
Tier I to III	• In principle, test results designated for Tier III would be required: Test results of genotoxicity and sub-chronic toxicity.
Tier IV	• In principle, test results designated for Tier IV would be required: Test results of genotoxicity, sub-chronic and chronic toxicity, carcinogenicity, reproductive and developmental toxicity, and ADME.
Mixture of chemical substances	• In principle, the data requirement would be same with the case for Metals, Inorganic compounds, Proteins. • However, if enough evidence indicate for no inclusion of substances that are required special consideration, data requirement can be the same as in normal tiered approach.
Nanomaterials	• The data requirement is case by case basis, because of possible differences on toxicological characteristics with substances that are not produced by new technology.

## 5. Exposure Assessment

In principle, “DC (mg/kg diet)” calculated in section 3 is converted to the estimated daily exposure (mg/kg bw/day) by formula 4 with the information on food consumption (kg/day) and body weight (kg) of the subject population.

$$\begin{array}{c} \text{Estimated daily exposure}\left(\text{mg}/\text{kg bw}/\text{day}\right)\\ =\frac{\text{DC mg}/\text{kg diet}\times \text{Food consumption}（\text{kg}/\text{day}）}{\text{Body weight}（\text{kg bw}）} \end{array}$$ (Formula 4)

The average values of Japanese population are basically used for the calculation of the food consumption and the body weight. If human populations that are highly sensitive to the exposure and the resulting toxicity are supposed, the exposure should be estimated in the populations, using relevant data such as National Health and Nutrition Survey by the MHLW.

## 6. Risk Characterization

In the step of risk characterization, the results of toxicity assessment (section 4) and exposure assessment (section 5) are integrated. The principal approach for risk characterization is different for each Tier of DC, because the data requirement on toxicities is different for each of them (Table 10). When Tier of DC is Tier I or II, risk characterization is conducted based on the available information or test results for genotoxicity in principle. When Tier of DC is Tier III or IV, the health risk for the subject human population is characterized by comparing the estimated daily exposure with HBGV (e.g. ADI, TDI) or Point of Departure (POD) (e.g. NOAEL) for the subject substance.

**Table 10.** The principal approach for risk characterization in each Tier of DC

Tier I or II	
Genotoxic substance	Intentionally used substances as raw materials of ACP • The use of the substances shall be judged not to be acceptable in principle. Unintentionally contained substances in the materials of ACP • The necessity of usage restriction of raw materials from which the substances are derived shall be assessed considering the information or the data related to the substances comprehensively.
Non-genotoxic substance	• The health risk shall be estimated to be low enough because the concern for non-carcinogenicity and non-genotoxic carcinogenicity will be low when the exposure level is below or at upper limit of Tier I or II.
Tier III	
Genotoxic substance	Intentionally used substances as raw materials of ACP • The use of the substances shall be judged not to be acceptable in principle. Unintentionally contained substance in the material of ACP • The necessity of usage restriction of raw materials from which the substances are derived shall be assessed considering the information or the data related to the substances comprehensively.
Non-genotoxic substance	The case where HBGV (e.g. ADI, TDI) is set • When the estimated daily exposure is below or at the HBGV, the health risk can be estimated to be low enough. • When the estimated daily exposure is above the HBGV, the condition of usage restriction or other relevant special attention shall be considered, because the health risk cannot be estimated to be low enough. The case where HBGV (e.g. ADI, TDI) need not to be set • The health risk shall be estimated by the Margin of Exposure (MOE) that is calculated from the POD (e.g. NOAEL) and the estimated daily exposure. • When the POD is determined based on the sub-chronic toxicity test result and the MOE is above or at approximately 100 – 1,000, the health risk can be estimated to be low enough. • For comprehensive consideration on the health risk, the reason why HBGV need not to be set and the magnitude of estimated daily exposure are also considered.
Tier IV	
Genotoxic carcinogen	Intentionally used substances as raw materials of ACP • The use of this substance shall be judged not to be acceptable in principle. Unintentionally contained substance in the material of ACP • The health risk shall be comprehensively assessed based on MOE approach. • When the MOE is above or at approximately 10,000, the health risk can be estimated to be low enough. • When the MOE is not enough large, the condition of the usage restriction or other relevant special attention shall be considered, because the health risk cannot be estimated to be low enough.
Non-genotoxic carcinogen	The case where HBGV (e.g. ADI, TDI) is set • When the estimated daily exposure is below or at the HBGV, the health risk can be estimated to be low enough. • When the estimated daily exposure is above the HBGV, the condition of usage restriction or other relevant special attention shall be considered, because the health risk cannot be estimated to be low enough. The case where HBGV (e.g. ADI, TDI) need not to be set • The health risk shall be estimated by MOE that is calculated from the POD (e.g. NOAEL) and the estimated daily exposure. • When the POD is determined based on the toxicity test result and the MOE is above or at approximately 100, the health risk can be estimated to be low enough. • For comprehensive consideration on the health risk, the reason why HBGV need not to be set and the magnitude of estimated daily exposure are also considered.

## 7. Future

As mentioned above, the Guidelines prescribe the principal approach for the risk assessment of new FCMs. When the assessment of them is requested by the MHLW, the FSCJ will carry out it on the basis of the Guidelines. The Guidelines also prescribe the condition for its revision. When the expert committee for ACP update the risk assessment approach on migration testing or toxicity tests, corresponding to the development of science, the international trend of risk assessment, or regulatory change of ACP in Japan, the Guidelines will be revised as necessary.

## Disclaimer Notice

The views and opinions expressed in the paper are those of the author and should not be attributed to the FSCJ.
